# Assessing the role of dispersed floral resources for managed bees in providing supporting ecosystem services for crop pollination

**DOI:** 10.7717/peerj.5654

**Published:** 2018-09-27

**Authors:** Annalie Melin, Mathieu Rouget, Jonathan F. Colville, Jeremy J. Midgley, John S. Donaldson

**Affiliations:** 1 Kirstenbosch Research Centre, South African National Biodiversity Institute, Cape Town, South Africa; 2 Statistics in Ecology, Environment and Conservation, Department of Statistical Sciences, University of Cape Town, Cape Town, South Africa; 3 Department of Biological Sciences, University of Cape Town, Cape Town, South Africa; 4 UMR PVBMT, CIRAD, La Réunion, France

**Keywords:** Managed pollinators, Beekeeper, Landscape availability, Honeybees, *Apis mellifera capensis*, Cape managed honeybee system, Eucalypt, Natural vegetation, Canola

## Abstract

Most pollination ecosystem services studies have focussed on wild pollinators and their dependence on natural floral resources adjacent to crop fields. However, managed pollinators depend on a mixture of floral resources that are spatially separated from the crop field. Here, we consider the supporting role these resources play as an ecosystem services provider to quantify the use and availability of floral resources, and to estimate their relative contribution to support pollination services of managed honeybees. Beekeepers supplying pollination services to the Western Cape deciduous fruit industry were interviewed to obtain information on their use of floral resources. For 120 apiary sites, we also analysed floral resources within a two km radius of each site based on geographic data. The relative availability of floral resources at sites was compared to regional availability. The relative contribution of floral resources-types to sustain managed honeybees was estimated. Beekeepers showed a strong preference for eucalypts and canola. Beekeepers selectively placed more hives at sites with eucalypt and canola and less with natural vegetation. However, at the landscape-scale, eucalypt was the least available resource, whereas natural vegetation was most common. Based on analysis of apiary sites, we estimated that 700,818 ha of natural vegetation, 73,910 ha of canola fields, and 10,485 ha of eucalypt are used to support the managed honeybee industry in the Western Cape. Whereas the Cape managed honeybee system uses a bee native to the region, alien plant species appear disproportionately important among the floral resources being exploited. We suggest that an integrated approach, including evidence from interview and landscape data, and fine-scale biological data is needed to study floral resources supporting managed honeybees.

## Introduction

Managed honeybees are globally important for crop production (Potts et al., 2010; Klein et al., 2007) and several studies have assessed the landscape requirements of managed honeybees (Henry et al., 2012a; Henry et al., 2012b; Naug, 2009; Rogers & Staub, 2013; Gallant, Euliss & Browning, 2014; Härtel & Steffan-Dewenter, 2014; Sponsler & Johnson, 2015). A critical component that has not been accounted for in these studies is the putative ecosystem services provided by natural and human-modified landscapes that support managed pollinators (Millennium Ecosystem Assessment, 2003; Costanza et al., 1997), especially if they are not contiguous with the crop field where pollination services are delivered. Such an assessment is essential if an integrated management approach to pollination services is to be adopted, considering both wild and managed pollinators (Garibaldi et al., 2013; Melin et al., 2014).

**Figure 1:**
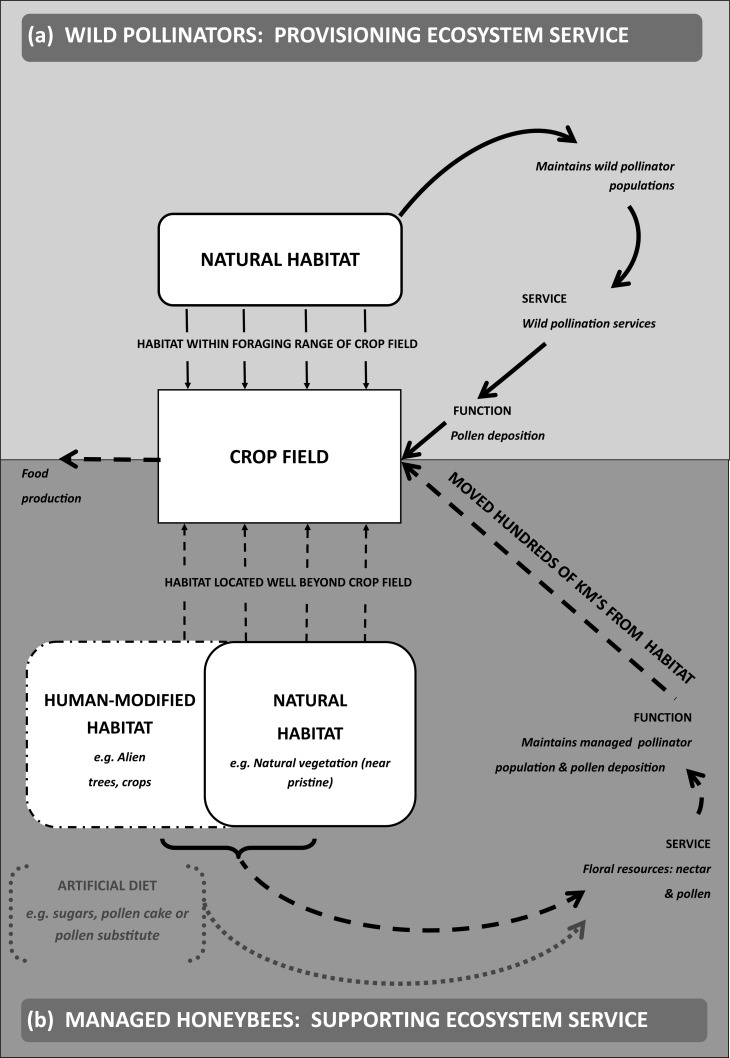
Supporting ecosystem services for managed pollinators. (A) shows the delivery of pollination ecosystem services within an agricultural landscape (adapted and simplified from Fig. 1 Kremen et al., 2007) depicted by solid black arrows. In contrast, (B) shows the ecosystem services supporting managed pollinators and how the landscape elements (natural and human-modified habitats (Millennium Ecosystem Assessment, 2003; Costanza et al., 1997) may be located well beyond the crop field which is depicted by wide-dashed arrows. Floral resource constraints for managed honeybees may be overcome with artificial feeding (e.g., sucrose, high fructose syrup, pollen cake) (Vanengelsdorp & Meixner, 2010; Keller, Fluri & Imdorf, 2005) and is depicted as narrow-dashed arrows.

Kremen et al. (2007) proposed a general conceptual model that has been used as a basis for understanding pollination ecosystem services at a landscape scale (see Kennedy et al., 2013; Lonsdorf et al., 2009). In essence, this model considers how mobile species, such as bees, utilise the landscape surrounding the crop and how the ecosystem services they provide are affected by land-use decisions within that landscape. The model applies over relatively short distances within spatially contiguous landscapes (Kremen et al., 2007). The inclusion of managed honeybees into this kind of model adds an additional dimension because landscape elements may not be contiguous with the farms where pollination services are provided. Honeybees are moved large distances by beekeepers due to the spatial and temporal separation of landscapes that have critical floral resources for bees and these may be separated from the crop fields where the pollination service is required (Gallant, Euliss & Browning, 2014; Melin et al., 2014). Here we examine the relative importance of natural and human-modified landscapes for supporting managed honeybees over spatial scales that extend well beyond the farm level (Fig. 1).

The existing literature supports the idea that natural vegetation is an important resource for managed honeybees and represents a key supporting ecosystem service to sustain honeybee populations outside the months when the crop is in flower (Hepburn & Radloff, 1998; Pirk et al., 2015; Naug, 2009). Despite its importance, natural vegetation may not provide a sufficient supply of pollen and nectar for managed honeybees throughout the year (Johnson, 1993; Hepburn & Guillarmod, 1991). This constraint may be overcome in agricultural landscapes where the temporal lack of floral resources in natural and semi-natural habitats can be compensated by the availability of other plants that have been introduced through human activity (e.g., crop plants, agricultural weeds). This is particularly true for honeybees that tend to seek out and utilise large, abundant patches of floral resources (Steffan-Dewenter & Tscharntke, 2000; Henry et al., 2012b; Visscher & Seeley, 1982). The availability of mass flowering crops (e.g., oilseed rape) in the landscape has been positively correlated with high densities of generalist pollinators (Westphal, Steffan-Dewenter & Tscharntke, 2003). Similarly, alien invasive plants that occur at relatively high density and abundance can be an important resource for pollinators (Williams et al., 2011). Notwithstanding the apparent benefits of diverse floral resources (Alaux et al., 2010a; Alaux et al., 2010b; De Groot, 1952), monofloral resources with a high nutritional value (e.g., protein rich pollen) may be comparable to polyfloral resources (Di Pasquale et al., 2013). As a result, even simplified human-modified landscapes can play a significant role in sustaining both wild and managed pollinators.

An important requirement when analysing the supporting ecosystem services provided by natural and human-modified landscapes is the spatial location of apiary sites and the factors that influence the use of apiary sites. Available floral resources delimit the landscape in which beekeepers operate, but there may be variation in how beekeepers manage their operational responses to maximise the long-term productivity of hives (Pellet, 1946; Vanengelsdorp & Meixner, 2010). These responses include the choice of apiary sites as well as the number of hives that are placed at a particular site (Johannsmeier, 2001). We consider here that the relative importance of floral resources supporting pollination services is influenced by a combination of the available landscape and the beekeeper’s operational response.

We examine managed honeybees in the context of the Western Cape (South Africa) where the endemic Cape honeybee (*Apis mellifera capensis*) provides important pollination services to agriculture, particularly to a significant deciduous fruit industry (Melin et al., 2014; Johannsmeier, 2001; Allsopp, De Lange & Veldtman, 2008). Within this area, the migratory beekeepers who provide pollination services utilise a range of spatially dispersed resources to sustain their hives, including natural vegetation and various human–modified landscapes, such as stands of eucalypt and canola crop fields.

Within this system, we specifically address the following questions:

1. Which floral resources do beekeepers consider most important for the long-term productivity and health of their hives?
2. Is beekeeper use consistent with availability of resources in the landscape?
3. What is the relative importance of natural vegetation versus human-modified landscapes for supporting ecosystem service for managed pollinators?

To answer these questions, we used a socio-ecological approach and collected data from beekeepers regarding their choice of locations for apiary sites and then undertook site surveys and spatial analyses of landscape features to determine the availability of floral resources at apiary sites. The relative availability of resources at apiary sites could then be compared to the availability of resources in the region.

## Methods

### Study system

The Cape managed honeybee system in South Africa can be defined by the biogeographic distribution of the Cape honeybee (*Apis mellifera capensis*) (Fig. S1, Hepburn & Crewe, 1991; Hepburn & Guillarmod, 1991; Hepburn & Radloff, 1998). This endemic species has adapted to the local environmental conditions and co-evolved with the native flora. Its distribution predominantly overlaps with the floristically rich Fynbos biome but also includes some areas of the Nama-karoo, Succulent Karoo, Albany Thicket and Forest Biomes (Mucina & Rutherford, 2010). Within this system, we focused on the Western Cape deciduous fruit industry, an industry valued at US$ 688 million per year (Greef & Kotze, 2007; Hortgro, 2012; Melin et al., 2014) and that is largely dependent on managed honeybees for pollination services (Allsopp, De Lange & Veldtman, 2008; Melin et al., 2014). This service is supplied by an estimated 30,000 managed hives (Allsopp & Cherry, 2004). Within this system, beekeepers move their hives over hundreds of kilometres to access floral resources (for honey production, comb build-up, overwintering and swarm trapping) or to deliver pollination services. They utilise a range of floral resources, including natural vegetation (fynbos), stands of introduced trees (eucalypt), agricultural weeds (e.g., *Echium plantagineum*) and cultivated crops (e.g., canola, citrus, clover, and lucerne), and stock their apiary sites with between five and 180 hives, depending on the type and extent of floral resource being used. This study focused on three general floral resource-types (natural vegetation, eucalypt*,* and crops/canola, see Table 1) because they are considered most important by commercial beekeepers (Melin et al., 2014; Johannsmeier, 2001) and spatial data were available for these resource-types. These three floral resources become available over a temporal mosaic across the Western Cape. Eucalypt typically becomes available during summer months (November-February), when there is a shortage of alternative floral resources (see Fig. 1 in Melin et al., 2014). Fynbos, available all-year-round but fluctuates with rainfall-seasonality, is typically targeted in winter months (April–August). Canola typically flowers over the spring months (July–August). From year to year, the beekeeper may adjust the time hives spend at an apiary site depending on the quality of the floral resource and the requirements of the colony.

**Table 1:** Summary of floral resource-types. A summary of the three main floral resource-types, the source of the data used, and the habitat type category for each floral resource. We obtained information for floral resources from interviews with beekeepers and land cover type was derived from available spatial data when mapping BLUs.

**Floral resource**	**Targeted plants**	**Land cover type**	**Habitat type**
Natural vegetation	Indigenous plant species of succulent karoo, fynbos, and renosterveld vegetation	Flower-rich vegetation including plants known to be used by honeybees such as *Erica* species [17] Used National Vegetation Map [34]	Natural (near pristine)
Eucalypt	*Eucalyptus cladocalyx, E. camaldulensis, and* *E. conferruminata*	Eucalypt stands digitized from aerial photos	Human-modified (invasive alien trees)
Crops (beekeeper dataset)	Canola, citrus, clover and lucerne	–	Human-modified (crops)
Canola fields (landscape analysis)	*Brassica napus*	16% of arable field boundary data/layer [38]—see Methods S1	Human-modified (crops)

### Beekeeper interviews and mapping apiary sites

Beekeepers were selected for one-to-one interviews using purposive sampling (De Vaus, 2002), in which respondents were selected because they were recognised as large commercial beekeepers (managing more than 600 hives) who provide pollination services to the Western Cape deciduous fruit industry. Respondents were identified using a snowballing technique (De Vaus, 2002) whereby beekeepers interviewed were asked to recommend the next beekeeper. The aim was to obtain a geographically representative sample of 6,000 managed hives representing approximately 20% of all hives used for pollination services in the Western Cape Province.

Six beekeepers were interviewed between June 2012 and October 2013. During the interviews, each of the beekeeper’s apiary sites were mapped using digital topographic maps (obtained from National Geo-Spatial Information, South Africa) with a scale of 1:50,000 in ArcGIS 10.1 (ESRI, Redlands, CA, USA).

Semi-structured interviews (Milner-Gulland & Rowcliffe, 2007) comprising open-ended questions, were used to obtain the following operational data: total number of hives; GPS coordinates of each apiary site; number of hives at each apiary site; the floral resources used and availability of floral resources (flowering time).

### Assessment of floral resources that beekeepers indicated as being used

Information was obtained for 6,700 managed hives, representing approximately 22% of the managed hives in the Western Cape Province. Data for 708 apiary sites (ranging from 26 to 364 apiary sites per beekeeper) was captured across a wide geographic distribution of the Cape managed honeybee system (Fig. S1).

For each beekeeper, a random selection of 20 apiary sites (giving a total of 120 sites) was chosen for all further analyses. We tested whether the selected subset of apiary sites was representative of the full set of apiary sites by carrying out a Pearson’s chi-squared test. There was no significant difference in the proportion of resource-types between the full dataset and the subsample (*χ*^2^ = 0.29, *df* = 2, *p* = 0.86).

**Figure 2:**
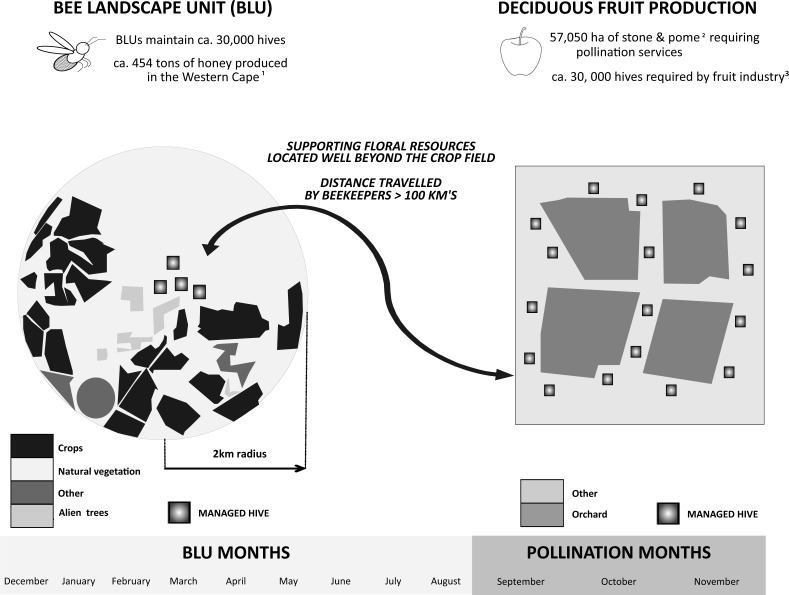
Bee landscape unit (BLU) and its separation from crop fields. Schematic diagram showing how the BLU is typically separated from the crop field where the provision of pollination services occurs. Each BLU is typically composed of a range of floral resources providing essential pollen and nectar which sustains managed honeybee populations. (Refs for the data in Fig. 2: ^1^ Allsopp & Cherry (2004), ^2^ Extracted from Western Cape AgriStats (2013),^3^ Melin et al. (2014).

### Bee Landscape Unit (BLU) definition and floral resource classification

The landscape surrounding each site (*n* = 120) was classified into a BLU (Fig. 2). A BLU is defined here as the vegetation within a two km radius of an apiary site. Each BLU comprised 1256 ha and we classified the available floral resources within this area into three categories (natural vegetation, eucalypt, and canola fields; Table 1). A radius of two km is consistent with other similar studies (Steffan-Dewenter & Kuhn, 2003; Gallant, Euliss & Browning, 2014; Henry et al., 2012b) and takes into account a range of factors that determine honeybee foraging distance, including honeybee health, complexity of the landscape, floral resource availability and climatic conditions (Gallant, Euliss & Browning, 2014; Steffan-Dewenter & Kuhn, 2003; Beekman & Rathnieks, 2000; Nguyen et al., 2009; Visscher & Seeley, 1982; Waddington & Holden, 1979; Ghazoul, 2005; Steffan-Dewenter & Tscharntke, 2000; Steffan-Dewenter, Münzenberg & Bürger, 2002). However, most of these studies were based in the Northern Hemisphere and were mainly inferred from indirect methods such as translocation experiments, mark recapture experiments or pollen analysis (see Table 1 in Zurbuchen et al., 2010). The radius of two km, therefore, may not be ideal for natural vegetation (e.g., fynbos) alone but it is a reasonable and logistically practical estimate. The BLUs were mapped in ArcGIS 10.1 using multiple spatial data sources and approaches (Methods S1). It was not logistically possible to ground-truth the large number of apiary sites in this study (*n* = 120) and over such an extensive area (>150,000 ha). However, we used expert knowledge about the landscape surrounding the apiary site from the beekeeper to guide the digitisation of floral resources.

In finalising the BLU classification, areas unlikely to provide pollen and nectar resources for honeybees such as major water bodies (e.g., dams) and all other agricultural types (e.g., viticulture) were excluded. In addition, the BLU analysis is unlikely to be very effective for assessing floral resource availability of weeds or private gardens (e.g., in urban areas and rural farmhouses) which are patchily distributed and because of the lack of suitable land cover data to estimate landscape availability.

#### Calculating regional-level floral resources

To calculate the average available floral resources across the study system (approximately 7,804,891 ha, 60% of the spatial extent of the Western Cape Province, Fig. S1), the total available area for each floral resource-type (natural vegetation, eucalypt, and canola fields) was estimated.

The total available area of natural vegetation and canola fields was determined from the same data and approach used to classify the BLUs. Versfeld, Le Maitre & Chapman (1998) estimate of invaded area of eucalypt for the Western Cape was used to calculate the total extent of eucalypt stands for the study system.

We estimated that the study system was composed of 66.28% natural vegetation, 2.99% canola fields, and 0.09% eucalypt. Similar to the BLU classification, the remaining land cover (approximately 30.64%) was excluded because it was composed either of water bodies and other agricultural types (e.g., viticulture) that is unlikely to provide pollen and nectar resources for honeybees or floral resources that lacked suitable spatial data (e.g., weeds or private gardens).

### Data analysis

The analyses were based on three datasets: first, a table giving the number of times (counts) a floral-resource category was specified by each beekeeper (beekeeper dataset; Data S1); secondly, a table giving area in hectares of floral resources potentially available to bees, cross-classified by beekeeper and floral-resource category (landscape dataset; Data S2); and thirdly, a table was derived from the area of available resource-type adjusted according to the number of hives associated with the apiary sites (hive adjusted dataset; Data S3).

#### Assessing proportional floral resource use

To illustrate the different proportional use of resource-types, bipartite networks were constructed from the contingency table for each dataset using the “bipartite” package (Dormann, Gruber & Fruend, 2008). All analyses were carried out using the software R (R Core Team, 2015). Bipartite networks are typically used to describe two-level ecological interactions e.g., seed–disperser, plant–pollinator and predator–prey systems (Dormann, Gruber & Fruend, 2008; Memmott, Waser & Price, 2004; Tylianakis, Tscharntke & Lewis, 2007). We use them here in a general network to visualize a beekeeper-floral resource system (sensu Memmott, Waser & Price, 2004) and to determine the use of floral resource-types across all beekeepers, quantifying if beekeeper use is consistent with landscape availability.

To determine whether the resources identified by beekeepers was consistent with what was available in the landscape and hive adjusted dataset, Pearson’s chi-squared tests were performed on the beekeeper dataset. This provided a test of whether the observed frequency of each floral resource-type was significantly different from expected. Expected values were based on: (i) proportions of all three floral resource-types derived from the landscape dataset (expected probabilities: eucalypt = 0.02, natural vegetation = 0.89 and canola = 0.09); (ii) the proportions derived from the hive adjusted dataset (expected probabilities: eucalypt = 0.89, natural vegetation = 0.02 and canola = 0.09) (McDonald, 2014; Abu-Bader, 2016).

To determine if BLUs differed in composition from what is generally available in the region and indicate whether beekeepers exhibit a preference for specific floral resources, the relative availability of floral resources across BLUs was compared to the average available floral resources across the whole study system. We did this by performing a nonparametric rank-sum test to determine whether the BLU sample mean of each floral resource-type differed significantly from its corresponding regional average using the “ICSNP” package (Nordhausen, Hannu Oja & Tyler, 2012). To produce the accompanying figure and calculate the 95% confidence intervals around the BLU sample means we used packages “Hmisc” (Harrell, 2015) and “latticeExtra” (Sarkar & Andrews, 2013).

#### Estimating the relative regional contribution of different floral resources

Using the total area of each floral resource category from the map of BLUs we estimated the relative contribution of natural and human-modified landscapes as resources for managed honeybee populations. We first divided the total area of each floral resource available within the BLUs by the total number of hives. Based on the required number of hives (approximately 30,000 managed hives) needed for pollination services for the Western Cape deciduous fruit industry, we estimated the supporting ecosystem service contribution of each floral resource. Although this estimate is a simple extrapolation from area and hive numbers there are currently a range of methods for mapping and measuring ecosystem services (Grêt-Regamey et al., 2015).

#### Compliance with ethical standards

Research ethics clearance involving human participants was granted by the Science Faculty Research Ethics Committee at University of Cape Town (UCT), reference number: SFREC 34-2012. In accordance with UCT’s Code of research involving human subjects, the nature of the research was verbally explained before each interview and each participant was provided with an information sheet outlining the research and provided contact details if concerns or questions arose following the interview. AM obtained signed consent from each of the participants prior to each interview. In all cases inputs were coded to retain the anonymity of the beekeeper and location of their sites and we only provide summary results in this paper.

## Results

### Floral resource use as indicated by beekeepers and BLU-analysis

**Figure 3:**
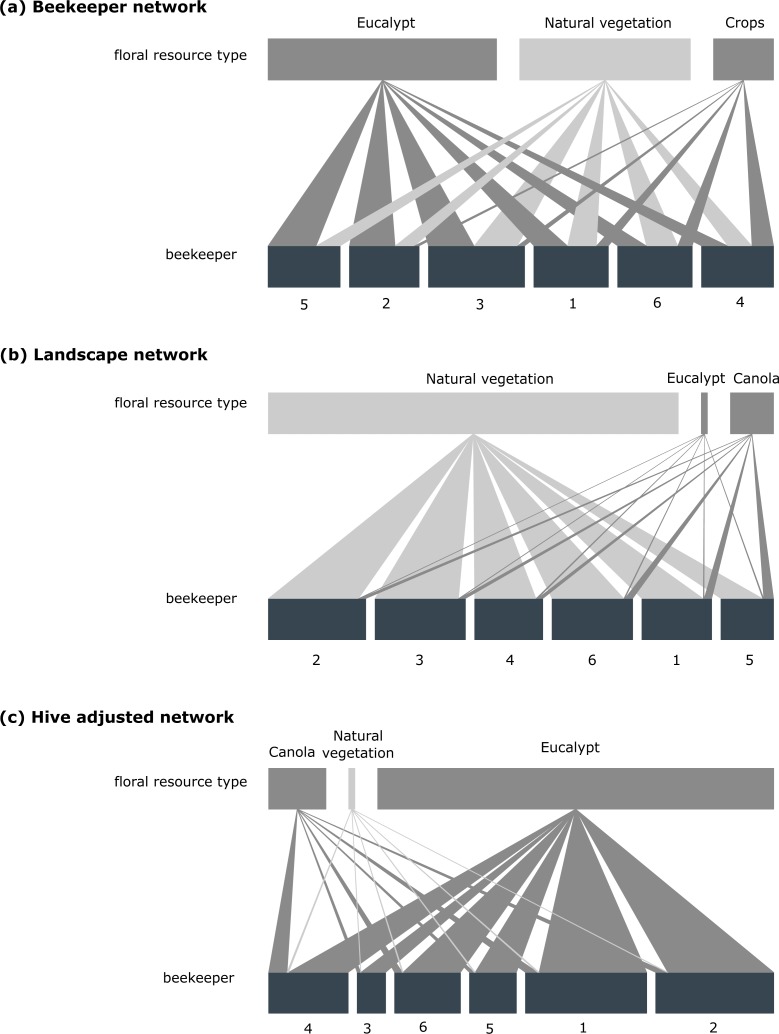
Beekeeper-floral resource networks. Importance of floral resource-types according to (A) the beekeeper dataset, (B) the landscape dataset (area in ha), (C) hive adjusted dataset (landscape availability adjusted by number of hives placed at apiaries). For (A) the floral resource-types and beekeepers are represented by rectangles (top and bottom of each graph). The lengths of the upper rectangles are proportional use of a resource-type considered across all beekeepers. The widths of the ties linking resource-types to beekeepers are proportional to the number of times a beekeeper used a particular resource-type. Interpretation for (B) and (C) is similar to (A), except that (B) is the area of a resource-type available to bees, rather than counts, and (C) is the area of a resource-type adjusted by hive number. Supporting ecosystem-services provided by natural vegetation are shown in light grey, whereas contributions provided by human-modified landscapes (in the form of eucalypt and crops) are shown in dark grey.

The network figure (Fig. 3A) shows clearly that beekeepers in the Cape managed honeybee system say they are using proportionally more eucalypts (50%), than natural vegetation (37%), and canola fields (13%) (Fig. 3A).

Analysis of the resources available to beekeepers within BLUs provided a significantly different perspective to that provided by beekeepers (*χ*2 = 2237.60, *df* = 2, *p* < 0.001). Eucalypt was the least available resource (mean across all beekeepers = 168 ha; range 116–264 ha), as they typically occur in small stands across the region whereas natural vegetation (mean across all beekeepers = 11,221 ha; range 6,855–14,806 ha) was most common (Fig. 3B).

**Figure 4:**
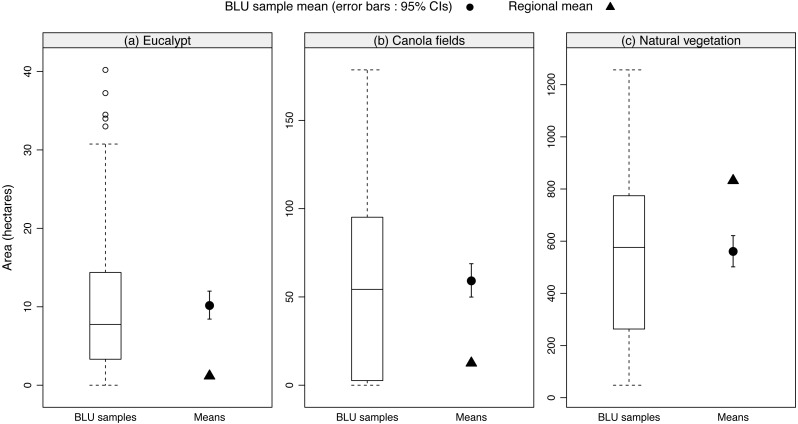
The relative availability of floral resources across BLUs compared to the regional average availability. Each panel represents the main floral resource-types (A–C). Within each panel, we plot the distribution of the data across the BLU samples. The BLU sample mean is shown as a circle and 95% confidence intervals were estimated using bootstrap methods (20,000 iterations). The estimated regional average is shown as a triangle. We interpret the position (above or below) of the BLU sample mean in relation to the regional average to determine if more or less hectares are being used across all beekeepers.

When the availability of different floral resources was adjusted according to the number of hives at an apiary site (hive adjusted dataset), eucalypt was found to be of primary importance (Fig. 3C). This finding was supported by the comparison of the BLUs with the regional average (*T* = 59.05, *p* < 0.001). This significant result was confirmed by Fig. 4A with the regional mean of eucalypt not falling within the confidence intervals of the BLU sample mean.

When analysing the beekeeper dataset against the hive adjusted dataset we find that these are significantly different (*χ*^2^ = 1218.10, *df* = 2, *p* < 0.001), particularly with regards to canola (Fig. 3C). As with eucalypt, beekeepers used significantly more canola (Fig. 4B, *T* = 59.05, *p* < 0.001) than the regional average.

In contrast, natural vegetation was used significantly less than the regional average despite it making up a large proportion (Fig. 3B) of the BLUs (*T* = 46.16, *p* < 0.001). When the landscape availability of natural vegetation was adjusted relative to hive number, its importance was reduced (Fig. 3C).

Based on the composition of BLUs, the population of managed honeybees in the Western Cape uses the following areas of floral resources: 700,818 ha of natural vegetation (13% of study system), 10,485 ha of eucalypt (0.2% of study system), and 73,910 ha of canola fields (1.4% of study system) (Table 2).

**Table 2:** Contribution of supporting ecosystem services. Estimate of the relative contribution of supporting ecosystem services provided by natural and human-modified landscapes to sustain the population of 30,000 managed honeybee hives in the Western Cape.

**Floral resources**	**Area (ha) within BLUs**	**Hectares per hive**	**Estimated area (ha)**	**Proportion of study system (%)**
Natural vegetation	67,325.22	23.36	700,818	13.0
Eucalypt	1,007.24	0.35	10,485	0.2
Canola fields	7,100.33	2.46	73,910	1.4

## Discussion

This paper focused on managed pollinators, which may be supported by diverse floral resources that are spatially dispersed across a broad region far away from the target crop field. In this situation, the different floral resources become connected through the actions of beekeepers and this has three important implications for the way ecosystem services for pollination are conceptualised and evaluated. First, it shifts the scale at which ecosystem services for pollination are evaluated from the target crop field to a larger geographical region. Second, it expands the type of ecosystem services being evaluated from provisioning services for pollination at the level of the crop field to include supporting services provided by floral resources for managed bees at the landscape and regional scale. Third, it means that the relative importance of different floral resources to support pollination may be influenced both by their availability in the region where managed bees occur as well as by operational responses of beekeepers to the available resources.

Several studies have used beekeeper surveys to identify floral resources (e.g., De Lange, Veldtman & Allsopp, 2013; Hutton-Squire, 2014; Masehela, 2017) but this study went further by comparing data from beekeeper interviews with data on the location of apiary sites relative to the availability of different vegetation types in the region. Our data and subsequent conclusions could be improved with the incorporation of biological data, e.g., honey or pollen sampling. Our results show that diverse floral resources, comprising crops, natural vegetation and alien eucalyptus trees are all important for supporting managed honeybees in the Western Cape of South Africa. However, the relative use of different floral resources by beekeepers was not consistent with what was available in the landscape. Apiary sites typically had a higher representation of eucalypts than was available in the region and more hives were located at apiary sites with eucalypts. This evidence of an operational response to the availability of eucalypts is consistent with the views expressed by beekeepers that eucalypts are vital for sustaining managed hives for pollination services (Melin et al., 2014) and confirmed the results of recent surveys of beekeepers (Hutton-Squire, 2014; Masehela, 2017; Melin, 2016).

The combination of beekeeper surveys and field studies highlights the value of using more than a single proxy measure (such as beekeeper surveys) to determine floral resources. Data on the number of hives in relation to the available floral resources provided critical information on beekeeper operational responses. The ‘hive adjusted’ dataset provided the most informative results in that it provided a more nuanced measure of what the beekeepers said relative to what was available in the landscape.

### Floral resources required to support managed pollination services

One rationale for studies of ecosystem services is to identify and evaluate those natural assets that are required to provide services to people (Millennium Ecosystem Assessment, 2003; Daily, 1997) and various models and studies of pollination services have highlighted the value of natural vegetation adjacent to crop fields (Ricketts et al., 2008; Kennedy et al., 2013; Garibaldi et al., 2011; Kremen et al., 2007). Assessing the services required to support managed honeybees can illustrate the value of landscapes that are not contiguous with crops. This study showed that the largest estimated floral resource area was natural vegetation, which also formed a major part of the floral resource mix in BLUs. Nevertheless, results of surveys showed that beekeepers placed less value on natural vegetation and field data showed there were fewer hives at apiary sites where natural vegetation predominated. Beekeepers value eucalypts for honey production and canola as it allows colonies to build-up strength prior to pollination services (Melin et al., 2014). Beekeeper operational responses reinforced the view that natural vegetation can support fewer hives than alternative floral resources. Baseline data on the carrying capacity of natural vegetation to support managed honeybees for pollination services in unknown. Floral abundance within natural vegetation, such as fynbos, fluctuates seasonally (Johnson, 1993; Hepburn & Guillarmod, 1991) and there may be relatively little flowering at certain times despite the large area of natural vegetation within BLUs. The general perception in the beekeeping literature is that natural vegetation, such as fynbos, is only marginally productive for commercial beekeeping in South Africa (Johannsmeier, 2001) but may be targeted for honey production (Johannsmeier, 2005; Melin et al., 2014). In the current mix of floral resources, natural vegetation plays a supplementary role when beekeepers have access to eucalypts but what is not clear from this study is whether natural vegetation provides critical resources at times of year when other resources are not available. This requires more detailed studies of bee behaviours at apiary sites (Hawkins et al., 2015; De Vere et al., 2017; Steffan-Dewenter et al., 2017). It should also be noted that in other systems where managed honeybees occur, natural vegetation may be more productive than fynbos (for beekeepers) so that supporting services provided by vegetation that is separated from crop fields should still be evaluated as an ecosystem service.

Human-modified landscapes played a significant role in sustaining a large number of hives. The relative importance of these different floral resources therefore needs to be considered when estimating the landscape components required to provide the supporting ecosystem services for managed honeybees. Our study could only give an approximate indication of the floral resource requirements to support managed pollination services and further refinement through fine-scale field-based research is needed to determine what honeybees are using in the landscape (e.g., see Russo et al., 2013 for detailed plant–pollinator phenological assessments at the landscape-level). The estimate for each floral resource-type could be improved by incorporating floral cover (e.g., relationship between plants size and the number of flowers) and the amount of nectar/pollen provided per flower per plant (Williams et al., 2011; Saifuddin & Jha, 2014). The flowers that honeybees choose to forage on may not correspond with the beekeeper’s choice. An analysis of pollen loads collected by honeybees would provide essential information on how honeybees are actually using the landscape and if beekeepers are making choices that allow honeybees to save energy by placing hives close to preferred floral resources.

Of particular interest is that the area of eucalypt needed to support the current number of hives for managed pollination services is estimated at a little over 10,000 ha. This is probably an under-estimate due to the exclusion of riverine species of eucalypt in BLUs*,* which could not be digitised with any confidence. The results are especially relevant to regulations regarding invasive *Eucalyptus* species and invasive species management programmes which coordinate ongoing clearing of eucalypts in the Western Cape Province (Van Wilgen et al., 2012).

It was surprising that beekeepers ranked crop floral resources (different to the target crop requiring pollination) as the least important resource. Beekeepers are thought to be shifting towards a greater use of crops such as canola to “boost” managed honeybee populations before hives are moved to farms for pollination (Melin et al., 2014) and the estimated use of canola fields (74,000 ha) was almost the same as the currently estimated area of production of the crop in the Western Cape (78,050 ha) (Crop Estimates Committee, 2015). However, beekeepers expressed concern about the use of pesticides on canola. This was also noted in other beekeeper surveys in the region (Allsopp & Cherry, 2004) and is supported by mounting scientific evidence that certain pesticides are harmful to bees (Pettis et al., 2013; Staveley et al., 2014; Nguyen et al., 2009; Van der Sluijs et al., 2015; Mao, 2013; Henry et al., 2012b).

An important question to consider in terms of overall sustainability is what would happen if particular “supporting” resources, such as eucalypt was removed or lost. Would this constrain crop production in the Western Cape or could hive numbers be sustained on other resources such as natural vegetation despite its seemingly lower carrying capacity for managed hives. It is not clear how and if beekeepers would adapt to changes in floral resource availability, i.e., would beekeepers switch to alternative resources or would such losses result in decreased profitability and ultimately closure of their business? Conducting detailed behavioural studies to investigate beekeeper’s responses to changing resource availability could provide insights into the future direction of the industry and how it relates to the provision of pollination services. The analyses in this study do not fully answer this question and it is clear that we are only beginning to understand the importance of different floral resources for managed honeybees in South Africa and other regions that are heavily reliant on managed pollinators.

The results of this study are not unique to the South African context. Many agricultural systems rely on managed honeybees (Klein et al., 2007; Potts et al., 2010) and beekeepers need to move their hives to access floral resources (e.g., Beekman & Rathnieks, 2000; Oldroyd, 2007), which may be separated from the crop field where they provide pollination (Gallant, Euliss & Browning, 2014). The ideas tested here therefore apply generally to systems where managed honeybees are important for crop pollination. As such, landscape models which seek to understand and provide a general framework for the conservation of pollination services, would need to consider the incorporation of floral resources away from the target crop.

Inclusion of supporting services brings in additional complexity to the management of ecosystem services for pollination because the managers of land where supporting services for pollinators are provided may not derive any direct benefit. In addition, the managed bees being supported at remote locations may provide pollination services to multiple crops. As a result, the management of floral resources for managed bees may benefit from studies of other ecosystem services such as water provision where there is a need to incentivise appropriate land management in water catchments to provide water to downstream users (Stosch et al., 2017).

An additional complexity that would need to be considered in landscape models incorporating managed honeybees is the importance of both natural and human-modified landscapes. One of the main conclusions of our study is that human-modified components of the landscape (eucalypt stands) can play an integral role in supporting managed honeybees (Melin et al., 2014; De Lange, Veldtman & Allsopp, 2013; Johannsmeier, 2001). Again, this result is not unique within the South African context but adds to a growing body of literature that shows how human-modified landscapes benefit both managed pollinators (Rollin et al., 2013; Sponsler & Johnson, 2015) and wild pollinators in the absence of natural vegetation (Samnegárd, Persson & Smith, 2011). How one balances the value of these “novel ecosystems” with conservation programmes (e.g., invasive species management programmes) is much debated but options for management of these systems are available (Murcia et al., 2014; Hobbs et al., 2014; Hobbs, Higgs & Harris, 2009; Hobbs, Higgs & Harris, 2014). In most studies to date, the main focus has been on contribution from natural habitats (Samnegaard et al., 2011). Some models using managed honeybees have also considered the importance of “indirect” ecosystem services provided by natural vegetation away from where the pollination service is delivered (Mouton, 2011). A critical result of our study, however, is the recognition that several different floral resources provided by natural and/or human-modified landscapes may be spatially dispersed across a region and the way in which beekeepers manage hives determines the landscape use of managed pollinators (Fig. 1).

## Conclusions

This paper improves our understanding of honeybee landscape ecology and the floral resources needed to support managed honeybees. Existing conceptual models focus on landscapes surrounding the target crop but this study showed how managed pollinators are moved around a greater area and the need to consider a complex range of factors such as: social factors (e.g., beekeeper’s preference), ecological factors (e.g., the type of floral resources available), spatial factors (e.g., the landscape distribution of apiary sites), behavioural (e.g., bee foraging) and biological (e.g., plant phenology). Incorporating such factors has important implications for managed honeybees because each floral resource-type requires its own set of management or conservation interventions. These should be incorporated in a broader understanding of how to sustain pollination services. Ultimately, the practical value of estimating the relative contribution of floral resources needed to sustain managed pollinators is that it could inform land managers in maintaining and conserving the current level of floral resources (see Gallant, Euliss & Browning, 2014) or increase the extent of available floral resources by creating suitable habitats (Kremen & Ostfeld, 2005). Since many systems across the world rely heavily on managed pollinators, and these pollinators are subject to similar types of beekeeper operational practices utilizing natural and human-modified landscapes away from target crop fields, the results presented here are an important first step towards recognising the need to expand current landscape ecosystem service models.

##  Supplemental Information

10.7717/peerj.5654/supp-1 Figure S1 The distribution of the Cape managed honeybee system in South Africa Map of Cape managed honeybee system including the Western Cape deciduous fruit growing areas (Extracted from Western Cape Department of Agricultural Department Aerial Commodity Census, 2013) and the distribution of apiary sites based on beekeeper subsample of 120 sites (using quarter degree squares (QDS) so that the exact location of the apiary sites cannot be identified).

10.7717/peerj.5654/supp-2 Methods S1 BLU Classification for each floral resource-type Detailed methods of the BLU classification for each floral resource-type: Natural vegetation, eucalypts and canola (see ‘Bee Landscape Unit (BLU) definition and floral resource classification’ in manuscript).

10.7717/peerj.5654/supp-3 Data S1 Beekeeper dataset Summary of data derived from interviews with beekeepers (see ‘Data analysis’ in manuscript).

10.7717/peerj.5654/supp-4 Data S2 Landscape dataset Summary of data derived from the available resource-types (see ‘Data analysis’ in manuscript).

10.7717/peerj.5654/supp-5 Data S3 Hive adjusted dataset Summary of data derived from the area of available resource-types adjusted according to the number of hives associated with the apiary site (see ‘Data analysis’ in manuscript).

## Additional Information and Declarations

### Data Availability

The following information was supplied regarding data availability: The raw data are provided in the Supplemental Files.
