# Supplementary figures and images for: Assessing the role of dispersed floral resources for managed bees in providing supporting ecosystem services for crop pollination
Source: PeerJ. 2018 Sep 27;6:e5654. doi: 10.7717/peerj.5654 (PMC6164548; doi:10.7717/peerj.5654)

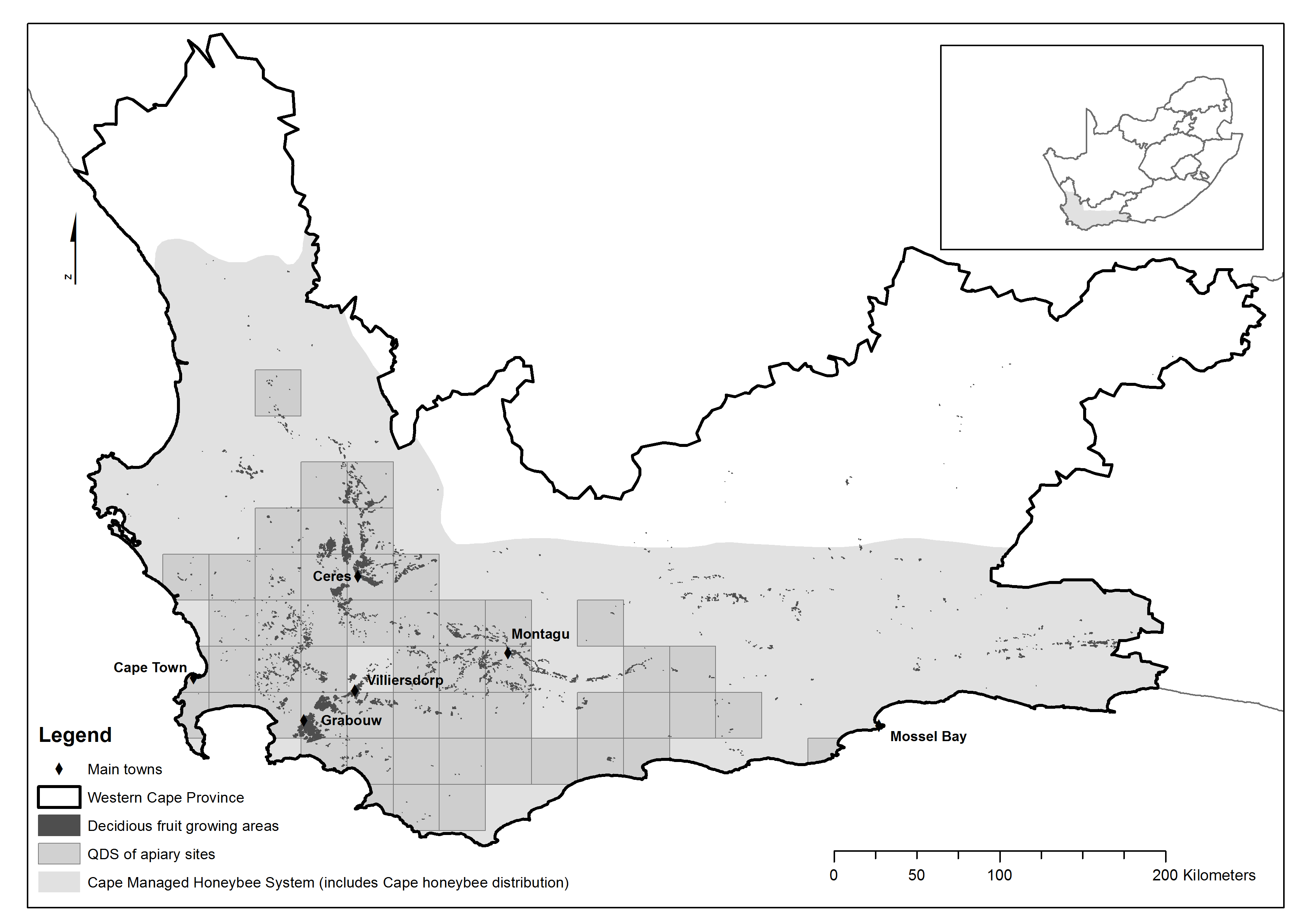

Supplement: Figure S1 — Map of Cape managed honeybee system including the Western Cape deciduous fruit growing areas (Extracted from Western Cape Department of Agricultural Department Aerial Commodity Census, 2013) and the distribution of apiary sites based on beekeeper subsample of 120 sites (using quarter degree squares (QDS) so that the exact location of the apiary sites cannot be identified). [file peerj-06-5654-s001.png]
