# Supplementary material for: Assessing the role of dispersed floral resources for managed bees in providing supporting ecosystem services for crop pollination
Source: PeerJ. 2018 Sep 27;6:e5654. doi: 10.7717/peerj.5654 (PMC6164548; doi:10.7717/peerj.5654)
Supplement: Methods S1 — Detailed methods of the BLU classification for each floral resource-type: Natural vegetation, eucalypts and canola (see ‘Bee Landscape Unit (BLU) definition and floral resource classification’ in manuscript). [file peerj-06-5654-s002.doc]

**Supplementary Information: Methods**

**BLU Classification for each floral resource-type (see Section 2.4 in manuscript)**

**Natural Vegetation**

In order to map the natural vegetation component of BLUs, the national vegetation map of South Africa (Mucina & Rutherford 2010) was used. Transformed areas, such as agriculture, were removed by overlaying the data layers on cultivated areas in the Western Cape (Department of Agriculture Forestry and Fisheries 2011).

**Eucalypts**

To estimate the spatial extent of stands of eucalypt, a combination of on-screen digitizing of colour aerial photographs and validation by beekeepers was used. The aerial photographs were obtained from National Geo-Spatial Information (South Africa) and were taken between 2008 and 2011 at a scale of 1:50 000. Eucalypt was relatively easy to distinguish from other resource-types, notably fynbos, because fynbos species are characterized by low-growing shrubs whereas eucalypts tends to grow in taller wood-lots surrounding or near homesteads. Mapping eucalypt in riparian areas could not be done because it was not possible to distinguish it with certainty from other invasive alien trees, e.g. Australian black wattle (*Acacia mearnsii*) (Versfeld et al. 1998). Unfortunately, there are no available estimates of the relative amounts of eucalypts in riparian areas for the Western Cape. Only 21 of our 120 sites (17.5%) included some riparian vegetation.

**Canola fields**

As there was no single source of spatial for canola because it is grown in rotation with other annual crops (e.g. wheat, barley and lucerne). This means that canola is unlikely to be grown in the same field in consecutive years. To estimate the canola component of each BLU, we first overlaid the map of cultivated areas in the Western Cape (Department of Agriculture Forestry and Fisheries 2011) in order to estimate the total area of arable land. The proportion of canola grown within the arable land was estimated by ground-truthing canola fields occurring within a 2 km radius of 20 apiary sites across the Western Cape in August 2013 during peak flowering. This provided an estimate that 16% of arable land was planted with canola and we applied this 16% rule for cultivated fields occurring in the BLUs.

**References**

Department of Agriculture Forestry and Fisheries, 2011. Field boundaries of cultivated areas in the Western Cape data layer.Western Cape, South Africa.

Mucina, L. & Rutherford, M.C., 2010. *The vegetation of South Africa, Lesotho and Swaziland*, Strelitzia 19. South African National Biodiversity Institute, Pretoria.

Versfeld, D.B., Le Maitre, D.C. & Chapman, R.A., 1998. *Alien Invading Plants and Water Resources in South Africa: A Preliminary Assessment. Report No. TT 99/98.* South Africa
